# Child sexual abuse and COVID-19 pandemic: another side effect of lockdown in Morocco

**DOI:** 10.11604/pamj.2021.38.57.27385

**Published:** 2021-01-18

**Authors:** Nour Mekaoui, Hanae Aouragh, Youssef Jeddi, Houda Rhalem, Badr Sououd Benjelloun Dakhama, Lamya Karboubi

**Affiliations:** 1 Paediatric Medical Emergency Department, Rabat Children's Hospital, Rabat, Morocco,; 2 Laboratory of Biostatistics, Clinical Research and Epidemiology, Faculty of Medecine and Pharmacy, Mohammed V University of Rabat, Rabat, Morocco

**Keywords:** Child abuse, COVID-19, pandemic, lockdown, Morocco

## To the editors of the Pan African Medical Journal

The World Health Organization has declared SARS-CoV-2 (COVID-19) a pandemic in March 11, 2020 [1]. The Kingdom of Morocco declared a state of health emergency from March 20 to June 24, 2020 and strict containment measures have been implemented including schools closure and suspension of extra-curricular activities [2]. While the number of pediatric medical emergency consultations has significantly decreased [3] during that period, we have seen an increase in the number of child abuses in our consultation. Social isolation is known to be a risk factor for child abuse [4,5]. Researchers have found that all types of child abuse become more common during school vacations, summer vacations, and natural disasters (epidemics, hurricanes, etc.) [4]. Thus, containment measures taken to halt the spread of SARS-CoV-2 made us expect an increase in cases of child abuse. We collected data of children under the age of 18 who were sexually abused attending the Pediatric Medical Emergency Department of the Children´s Hospital of Rabat (Morocco) during the lockdown period of March 30 to June 2020.

Fourteen children who were victims of sexual abuse consulted the pediatric medical emergency department; this number is 2.3 times the number who consulted during the same period in 2019. Although the rate is increased by 2.3, it certainly remains underestimated due to the difficulties by families of accessing hospitals and social structures [3]. Sexual violence mostly concerned children under 13 years old, seven victims were under five years of age and male victims represented half of the cases seen. The parental couple and the extended family were most often responsible for the cases of child abuse: ten of the victims reported a predominance touching compared to sexual penetrations. The physical examination was unremarkable in 70% of the abused children. Thirty percent presented either a digestive symptomatology or an abnormality on the anogenital examination. Sexual assault on minors in the family environment is a frequent phenomenon and is still by far underestimated in our country. The number of cases of child sexual abuse increased dramatically during confinement compared to the same period last year, despite difficult access to hospitals. The child is isolated, often confined with the potential perpetrator. Reporting systems for abused children are not optimal in our context and require us to introduce more measures for active screening for sexual assaults on minors in our context.
